# Polymer Membranes in Separation Process

**DOI:** 10.3390/membranes11110831

**Published:** 2021-10-28

**Authors:** Malgorzata Ulewicz

**Affiliations:** Faculty of Civil Engineering, Czestochowa University of Technology, Dabrowskiego 69 Street, PL 42-201 Czestochowa, Poland; malgorzata.ulewicz@pcz.pl; Tel.: +48-3-4325-0935

Among the many known physicochemical methods for the separation of organic compounds and metal ions from aqueous solutions, liquid–liquid extraction and membrane techniques hold a special position, enabling their recovery from dilute aqueous solutions. The liquid–liquid extraction process has an established position as a classic separation method for the removal and concentration of organic compounds and metal ions from aqueous solutions. Nevertheless, recently, liquid membranes—in particular polymer inclusion membranes—have enjoyed great interest from scientists around the world. Polymer inclusion membranes are formed by pouring a solution consisting of a mixture of a polymer, a plasticizer and a carrier. The synthesis of new and effective organic compounds used as carriers of organic compounds or metal ions influences the intensive development of polymer inclusion membranes, which are distinguished by their significant durability and chemical resistance, as well as their mechanical strength and stability. An important quality of polymer inclusion membranes is the possibility of modifying their composition. This is very important, because the synthesis of membranes with optimal compositions allows the achievement of efficiency and selectivity in the separation of both organic compounds and metal ions. The separation properties, as well as the efficiency and selectivity of the pertraction, depends not only on the composition of the membrane, but also on a number of other factors—including the aqueous feed and receiving phases’ composition. Determining the optimal conditions for separation of organic compounds or metal ions requires a lot of experimental research in order to improve the efficiency and selectivity of the transport processes. Modification of polymer membrane composition presents a real opportunity for increasing the possibility of their potential use in the future.

Within this special issue of “Polymer Membranes in Separation Process”, there are published the latest results of scientific research on the use of polymer membranes in the separation process—especially metal ions. Research in this field is being carried out in many centers and universities around the world, including Turkey, China, the USA, Malaysia, and Poland.

This issue contains six original research papers and one review article. The topics of these articles cover many aspects of the use of polymer membranes, including the separation of metal ions, recovery of metals from water and kinetics studies of the transport process of chemical compounds across membranes. Interesting and practical solutions, as well as the results of laboratory tests, are presented. Seval et al. [1] presented the separation and recovery of boron from geothermal waters in selective transport across the polymeric membrane system; this system can be easily used in industrial-scale applications for the removal of boron from geothermal water. Soo et al. [2] used polymer membranes with bis-(2-ethylhexyl) phosphate (B2EHP) to remove toxic colorants in the textile industry; the conditions for an effective colorant removal process as well as for the influence of membrane morphology on transport processes were defined in this study. Konczyk and Ciesielski [3] presented the facilitated transport of Pb(II) through polymer inclusion membranes (PIMs) containing 1,8,15,22-tetra(1-heptyl)-calixresorcin [4] arene and its tetra- and octa-substituted derivatives containing phosphoryl, thiophosphoryl or ester groups as ion carriers. The authors determined the dependence of transport of metal ions on parameters such as the membrane, feed and stripping phase composition, and temperature of the conducted process. Moreover, they presented kinetic models and optimal conditions for competitive transport of Pb(II) by PIMs from solutions containing Zn(II), Cd(II) and Cr(III) ions. Pyszka and Radzyminska-Lenarcik [4] determined the conditions of Zn(II) separation from solutions containing non-ferrous metal ions, such as (Co(II), Ni(II) Cu(II), Cd(II)), using polymer inclusion membranes with ethylenediamine-bis-acetylacetone as a carrier. The authors showed that the metal ion recovery rate strictly depends on the solution composition. Additionally, Gajda et al [5] demonstrated the possibility of separating non-ferrous metal ions via transport across polymer inclusion membranes containing derivatives of 1-alkyl-1,2,4-triazoles. The authors determined the conditions of Cu(II), Co(II) and Ni(II) ion separation through the membrane and showed that the transport rate of these metals depends on the membrane viscosity and the stability of the metal complexes with the ion carrier. In turn, Radzyminska-Lenarcie et al. [6] determined the separation conditions of Zn(II), Cr(III), and Ni(II) ions during transport across polymer membranes containing thylenodiamino-bis-acetylacetone. Both the conditions for an effective metal ion separation process as well as the influence of membrane morphology on the transport process have been defined in this study. This special issue is completed by a review article by Ulewicz and Radzyminska-Lenarcik [7]. This review covers the applications of polymer inclusion membranes containing alkylimidazole derivatives as carriers in the process of transporting ions of heavy and toxic metals, such as Zn(II), Cu(II), Cd(II), Co(II), Ni(II), and Mn(II). Alkylimidazoles are effective metal ion carriers as they exhibit various complexing properties with non-ferrous metal ions, and their properties (hydrophobic and alkaline) can be easily modified.

All the articles published in this special issue have been reviewed by recognized experts in the relevant fields of science. As Guest Editor, I would like to acknowledge all the authors for their valuable contributions. I would also like to thank the reviewers for their comments and suggestions, which have greatly improved the quality of the papers. Last, but not least, I would also like to thank the Section Managing Editor, Ms. Lydia Li, for her kind assistance in the preparation of this special issue of the journal.

## Data Availability

Not applicable.
